# Pharmacokinetics of doxorubicin co-administered with high-dose verapamil.

**DOI:** 10.1038/bjc.1995.27

**Published:** 1995-01

**Authors:** M. Gigante, G. Toffoli, M. Boiocchi

**Affiliations:** Division of Experimental Oncology 1, Centro di Riferimento Oncologico, Aviano, Italy.

## Abstract

The potential for the modification of the pharmacokinetics of doxorubicin (DOX) concurrently administered with high-dose verapamil (VER) has been investigated in 17 patients with advanced neoplasms refractory to drugs belonging to the multidrug resistance spectrum. Steady-state concentration of DOX, systemic clearance and urinary excretion were analysed. No significant difference was found between the kinetic parameters estimated for DOX at different levels of VER and those reported for doxorubicin as single agent. It can be concluded that VER does not appear to modify DOX kinetics.


### Pharmacokinetics of doxorubicin co-administered with high-dose verapamil

M Gigante, G Toffoli and M Boiocchi

*Division of Experimental Oncology 1, Centro di Riferimento Oncologico, Aviano (PN), Italy*

**Summary** The potential for the modification of the pharmacokinetics of doxorubicin (DOX) concurrently administered with high-dose verapamil (VER) has been investigated in 17 patients with advanced neoplasms refractory to drugs belonging to the multidrug resistance spectrum. Steady-state concentration of DOX, systemic clearance and urinary excretion were analysed. No significant difference was found between the kinetic parameters estimated for DOX at different levels of VER and those reported for doxorubicin as single agent. It can be concluded that VER does not appear to modify DOX kinetics.

**Keywords:** doxorubicin; MDR; pharmacokinetics; verapamil

Experimental data suggest that multidrug resistance (MDR) in cancer may be overcome by using anti-cancer agents in combination with resistance-modifying agents (RMAs) such as verapamil (Tsuruo *et al.*, 1983). Recently we demonstrated that the best results in overcoming *in vitro* MDR to DOX require continuous VER exposure, at concentrations $\geqslant 1\mu$M and for a time roughly similar to the cells' replication time (Boiocchi and Toffoli, 1992; Toffoli *et al.*, 1993). Based on these *in vitro* results, we are trying to design clinical trials based on a continuous infusion of DOX and VER, which may be more effective than previous clinical trials. For this purpose, knowledge of the consequences of the administration of RMAs on drug kinetics is fundamental. High-dose cyclosporine, for example, produces significant increases in etoposide systemic exposure (Lum *et al.*, 1992). Results concerning the kinetics of DOX modulated by VER are controversial (Kerr *et al.*, 1986). The purpose of the present study was to verify the possible variation in the kinetics of DOX concurrently administered with VER, infused at escalating dose rates throughout the DOX infusion. Escalating dose rates of VER infusion were planned to prevent sudden cardiovascular toxicity resulting from the required, high VER concentration. The prolonged infusion of DOX was selected because it offers, at least *in vitro* (Lai *et al.*, 1991), the possibility of increasing tumoricidal effects based on neoplasms expressing P-glycoprotein (P-gp), such as colon carcinoma. DOX at 75 mg m$^{-2}$ was infused over 96 h (schedule A) or 48 h (schedule B). This latter schedule was designed to achieve the target steady-state concentration ($C_{ss}$) of DOX at the beginning of the infusion, simultaneously with higher dose rates of VER infusion.

### Materials and methods

### *Patients*

Seventeen subjects, eight males and nine females, were included in this study. Median age was 42 years, ranging from 21 to 67. Primary sites were colon (13 patients), rectum (one patient), bone (one patient) and soft tissue (two patients). No patient had congestive heart failure and none had renal or liver dysfunction according to protocol eligibility criteria. The therapeutic protocol was approved by an ethical committee; the patients were informed of the nature of the study and gave consent for the treatment protocol.

Correspondence: M Boiocchi, Division of Experimental Oncology 1, Centro di Riferimento Oncologico, via Pedemontana Occidentale 12, 33081 Aviano (PN), Italy
Received 23 March 1994; revised 9 August 1994; accepted 11 August 1994

### *Administration of drugs*

VER (Isoptin, Knoll) administration by a central vein catheter started with a loading dose of 0.15 mg kg$^{-1}$ (time 0) and continued for 132 h. The infusion schedule was as follows: 0.2, 0.25, 0.3, 0.35 and 0.4 mg kg$^{-1}$ h$^{-1}$ at intervals of 0-12 h, 12-36 h, 36-60 h, 60-84 h and 84-132 h respectively. DOX was co-administered following two schedules: (A) the dose of DOX was 75 mg m$^{-2}$ per course (from hour 12 to hour 108); (B) a loading dose of 29 mg m$^{-2}$ DOX was given at hour 60; subsequent maintenance infusion of 23 mg m$^{-2}$ day$^{-1}$ lasted 48 h, until hour 108. Blood samples to measure DOX concentration were withdrawn at the following times: schedule A, hours 12, 24, 48, 60, 72, 96, 108, 132 and 144; schedule B, hours 60, 66, 72, 84, 108, 132 and 144. Urine were collected at intervals of 12 h, from hour 0 to hour 156. At least five samples were withdrawn after infusion (from hour 108 to hour 168) in five patients following either schedule A or B. Eleven patients followed schedule A, four schedule B and two patients followed both schedules.

### *Drug assays*

Serum and urine DOX concentrations were measured by high-performance liquid chromatographic (HPLC) assay (Zanette *et al.*, 1990) with slight modifications. Daunorubicin was added to each sample as the internal standard. The efficiency of the extraction of DOX and daunorubicin averaged 80% and 85% respectively. The assay was linear to at least 10 ng ml$^{-1}$; interassay coefficient of variation ranged from 5% to 15% at different concentrations.

Serum concentrations of VER and nor-verapamil (nor-VER), an active RMA metabolite, were measured by HPLC assay (Salama *et al.*, 1989). Sampling of blood to measure VER and nor-VER concentration was performed in all patients, at least in the first course, at the following times: hours 0, 12, 24, 36, 48, 60, 72, 84, 108, 132, 144 and 156.

### *Pharmacokinetic analysis*

In order to study the system at steady state (SS), a linear one-compartment model was adopted. Rate of infusion ($k_o$) was calculated as:

$$K_o = C_{ss} \times Cl$$

where $C_{ss}$ is the target steady-state concentration ($M/V$) and Cl is the apparent systemic clearance ($V/t$) (mean value reported in the literature; Benet and Williams, 1990). Observed $C_{ss}$ was calculated in each patient as the mean concentration of DOX on the last day of infusion. No correlation was found between concentration and time. The mean $C_{ss}$ was the mean value of observed concentrations during the last day of infusion. The variability in $C_{ss}$ is reported in Table I. The area under curve (AUC) was calculated using the trapezoidal rule (data not shown).

The data do not represent the early distribution phase of DOX after the onset of therapy or the late elimination phase 2 or more days after infusion. On these grounds a one-compartment model, with zero-order input and first-order output, was identified a priori. Fitting of the model to the data was good for almost all courses. The coefficient of correlation between observed and calculated $C_{ss}$ ranged from 0.7 to 0.99; the number of observations for each course ranged from 6 to 14.

The distribution of DOX in the body is much faster than elimination (Speth *et al.*, 1988), thus the post-infusion curve should very quickly approach the elimination phase curve. Since the elimination of DOX is a first-order process and the reported, final half-life of elimination is 30 h, three samples were sufficient to estimate the constant of elimination (Eksborg *et al.*, 1985). Individual ranges of variation of $C_{ss}$ in different courses are reported (Table I) only for the patients who underwent several courses with schedule B, while most patients following schedule A performed only one course. PCNONLIN version 4.0 was the non-linear regression program used to estimate kinetic parameters defining the model.

### *Statistical analysis*

*t*-Tests for unpaired samples, with superimposable variability, were performed to compare estimates of kinetic parameters from groups of patients following different treatments.

### Results

The observed $C_{ss}$ was $23 \pm 7$ ng ml$^{-1}$ (range 15–34 ng ml$^{-1}$) for patients following schedule A ($C_{ss}$A) while the calculated $C_{ss}$ was 24 ng ml$^{-1}$ (Table II). The observed $C_{ss}$ for patients following schedule B ($C_{ss}$B) was $35 \pm 8$ ng ml$^{-1}$ (range 29–55 ng ml$^{-1}$) and calculated $C_{ss}$ was 30 ng ml$^{-1}$. Mean ($\pm$ s.d.) DOX concentrations of six patients for schedules A and B are shown in Figure 1. The variability of $C_{ss}$ within the first course and between different courses in patients following schedule B is reported in Table I. Estimates of apparent volume of distribution ($V$), half-life of elimination ($t_{\frac{1}{2}}$) and apparent systemic clearance (Cl), together with values reported from the literature are reported in Table II. $V_A$ was $21 \pm 5$ l kg$^{-1}$ (range 16–27), $V_B$ was $20 \pm 6$ l kg$^{-1}$ (range 17–31) and $V_L$ was 25 l kg$^{-1}$ (range 9–66); $t_{\frac{1}{2}A}$ was $20 \pm 8$ h (range 14–32), $t_{\frac{1}{2}B}$ was $23 \pm 5$ h (range 16–29) and $t_{\frac{1}{2}L}$ was 30 h (range 14–37); $Cl_A$ was $13 \pm 4$ ml min$^{-1}$ kg$^{-1}$ (range 10–21), $Cl_B$ was $13 \pm 2$ ml min$^{-1}$ kg$^{-1}$ (9–16) and $Cl_L$ was 13 ml min$^{-1}$ kg$^{-1}$ (range 8–16). The fractions of DOX excreted in the urine (fu) were $9 \pm 3$% (A) and $7 \pm 2$% (B). Individual parameters ranged from 3.7 to 11.2%. Estimates of our study fell within the range reported in the literature (fu $<$ 15%) (Speth *et al.*, 1990). Estimates of treatments A and B were compared with each other and with reported estimates (Benet and Williams, 1990): no significant difference ($P > 0.6$) was found.

On average, VER peak level was 1,650 ng ml$^{-1}$ (ranging from 620 to 2,560 ng ml$^{-1}$) and serum concentrations of nor-VER, a metabolite active as a chemosensitiser, were 590 ng ml$^{-1}$ (ranging from 210 to 960 ng ml$^{-1}$).

Cardiovascular side-effects were limited and rapidly reversible after the completion of VER infusion. Data referred to 15 courses performed in nine patients. Prolonged QT was observed in 15 courses (15/15); other side-effects were junctional rhythm (9/15), first-degree block (4/15) and second-degree block (1/15). No hypotension (mean arterial pressure $<$ 80 mmHg) or congestive heart failure was observed. No patient had hyperbilirubinaemia.

**Table I** Intra-patient variability of observed $C_{ss}$ (schedule B)

| *Patient no.* | *No. of courses* | *[DOX][a] first course (ng ml$^{-1}$)* | *[DOX][b] (ng ml$^{-1}$)* |
|---|---|---|---|
| 1 | 3 | $34 \pm 6$ | 26–34 |
| 2 | 2 | $50 \pm 13$ | 50–55 |
| 3 | 3 | $35 \pm 4$ | 22–35 |
| 4 | 1 | $30 \pm 3$ | 30 |
| 5 | 2 | $35 \pm 12$ | 35–43 |
| 6 | 1 | $29 \pm 2$ | 29 |

[a]Concentration observed on the second day (hour 84–108) of infusion of DOX: mean $\pm$ s.d. [b]Range of $C_{ss}$ in different courses.

### Discussion

The association of antineoplastic agents and RMAs requires investigation of the possible kinetic variations in the drugs resulting from their interaction. The few data available on the interaction between VER and anthracyclines are controversial. A significant increase in the half-life of elimination of DOX was found when DOX was co-administered with VER (oral dose), but other kinetic parameters related to elimination did not differ significantly (Kerr *et al.*, 1986). Scheithauer *et al.* (1993) reported a significant decrease in the half-life of epirubicin co-administered with D-VER; on the other hand,

**Figure 1** DOX concentrations in serum during and after 96 h (○) and 48 h (●) infusion. Each point represents the mean value ($\pm$ s.d.) for six patients.

**Table II** Mean steady-state concentration ($C_{ss}$) of DOX, kinetic parameters of patients following schedules A and B and parameters of DOX reported in the literature

| *Schedule* | *Dox time of infusion (h)* | *Patient no.* | *$C_{ss}$ Expected[a] (ng ml$^{-1}$)* | *$C_{ss}$ Observed (ng ml$^{-1}$)* | *$V$[b] (l kg$^{-1}$)* | *$t_{\frac{1}{2}}$[c] (h)* | *Cl[d] (ml min$^{-1}$ kg$^{-1}$)* |
|---|---|---|---|---|---|---|---|
| A[e] | 96 | 13 | 24 | $23 \pm 7$[f] | $21 \pm 5$[f] | $20 \pm 8$[f] | $13 \pm 4$[f] |
| B[e] | 48 | 6 | 30 | $36 \pm 8$ | $20 \pm 6$ | $23 \pm 5$ | $13 \pm 2$ |
| | | | | | 25[g] | 30 | 13 |
| | | | | | 9–66[h] | 14–37 | 8–16 |

[a]See Materials and methods. [b]Apparent volume of distribution. [c]Half-life of elimination. [d]Apparent systemic clearance. [e]A, 96 h DOX i.v. infusion and 132 h high-dose VER infusion; B, bolus plus 48 h DOX infusion and 132 h high-dose VER infusion. [f]Mean $\pm$ s.d. [g]Kinetic parameters of DOX reported in the literature (Bennet and Williams, 1990). [h]Range of values reported in several studies (Speth *et al.*, 1988).

no variation in epirubicin kinetics was reported when epirubicin was co-administered with VER (Mross *et al.*, 1993).

The results of the present study do not show significant variations in the kinetics of DOX when concurrently administered with VER. The unpaired data of this study were compared with each other and with data from the literature since ethical constraints prevented treatment of patients without VER. In fact, 13 of the 17 patients analysed had colon carcinoma, in which monochemotherapy with DOX has no activity (Booser and Hrtobagy, 1994). The unpaired data analysis not allow us to detect whether the large inter-individual variations in DOX pharmacokinetics we observed could mask small effects of VER on DOX pharmacokinetics, if any. Nevertheless the robustness of the conclusion of our study stands on the following features:

(1) Similar results were obtained from two different DOX dosage regimens (schedules A and B).
(2) VER concentrations were continuous (e.g. concentrations approaching $C_{ss}$) and relatively high, differently from previous treatments with multiple oral dose (Kerr *et al.*, 1986); this regimen appears to be suitable to draw a conclusion on the kinetic variation of DOX.
(3) This treatment was apparently well tolerated from the haemodynamic point of view. Cardiovascular side-effects were limited and reversible, so possible variations in the pharmacokinetic parameters owing to impaired haemodynamics were excluded.
(4) No borderline *P*-value was found comparing kinetic parameters.

In conclusion, VER does not appear to modulate DOX kinetics. These data should be considered in the design of further schedules combining DOX with VER and the evaluation of the response and toxicity determined by these treatments to overcome MDR.

**Acknowledgements**
This work was supported by the Associazione Italiana per la Ricerca sul Cancro and CNR Ricerca Finalizzata, Contract No. 92.02366.PF39, Milan, Italy. The authors gratefully acknowledge Dr S Frustaci (Division of Medical Oncology, Centro di Riferimento Oncologico, Aviano (PN), Italy), Dr D Fantin (Intensive Care Unit, Centro di Riferimento Oncologico, Aviano (PN), Italy), Dr G Nicolosi (Cardiology Department, Ospedale Civile, Pordenone, Italy), Dr O Vinante (Division of Oncology, Centro Oncologico Multizonale, 'Noale (VE), Italy), Dr M Leoni (Division of Medical Oncology, Ospedale 'S Maria delle Croci', Ravenna, Italy) and Dr Franco Monti (Division of Medical Oncology, Ospedale 'Infermi', Rimini (FO), Italy) for cooperation on the study and thank Dr P Tonel for her help with the manuscript.

### References

BENET LZ AND WILLIAMS RL. (1990). Appendix II, pharmacokinetic data. In *The Pharmacological Basis of Therapeutics*, Goodman GA, Rall TW, Nies AS and Taylor P (eds) p. 1715. Pergamon Press: New York.

BOIOCCHI M AND TOFFOLI G. (1992). Mechanism of multidrug resistance in human tumour cell lines and complete reversion of cellular resistance. *Eur. J. Cancer*, **28A**, 1099–1105.

BOOSER DJ AND HRTOBAGY GN. (1994). Anthracycline antibiotics in cancer therapy. Focus on drug resistance. *Drugs*, **47**, 223–258.

EKSBORG S, STRANDLER HS, EDSMYR F, NASLUND I AND TAHVANAINEN P. (1985). Pharmacokinetic study of i.v. infusions of adriamycin. *Eur. J. Clin. Pharmacol.*, **28**, 205–212.

LAI GM, CHEN YN, MICKLEYLA, FOJO AT AND BATES SE. (1991). P-glycoprotein expression and schedule dependence of adriamycin cytotoxicity in human colon carcinoma cell lines. *Int. J. Cancer*, **49**, 696–703.

LUM BL, KAUBISCH S, YAHANDA AM, ADLER KM, JEW L, EHSAN MN, HALSEY J, GOSLAND MP AND SIKIC BI. (1992). Alteration of etoposide pharmacokinetics and pharmacodynamics by cyclosporine in a phase I trial to modulate multidrug resistance. *J. Clin. Oncol.*, **10**, 1635–1642.

KERR DJ, GRAHAM J, CUMMINGS J, MORRISON JG, THOMPSON GG, BRODIE MK AND KAYE SB. (1986). The effect of verapamil on the pharmacokinetics of adriamycin. *Cancer Chemother. Pharmacol.*, **18**, 239–242.

MROSS K, HAMM K AND HOSSFELD DK. (1993). Effects of verapamil on the pharmacokinetics and metabolism of epirubicin. *Cancer Chemother. Pharmacol.*, **31**, 369–375.

SALAMA ZB, DILGER C, CZOGALLA W, OTTO R AND JAEGER H. (1989). Quantitative determination of verapamil and metabolites in human serum by high-performance liquid chromatography and its application to biopharmaceutic investigations. *Drug Res.*, **39**, 210–215.

SCHEITHAUER W, SCHENK T AND CZEJKA M. (1993). Pharmacokinetic interaction between epirubicin and the multidrug resistance reverting agent D-verapamil. *Br. J. Cancer*, **68**, 8–9.

SPETH PAJ, VAN HOESEL QGCM AND HAANEN C. (1988). Clinical pharmacokinetics of doxorubicin. *Clin. Pharmacokin.*, **15**, 15–31.

TOFFOLI G, TUMIOTTO L, GIGANTE M, DALL' ARCHE MG, PERIN T AND BOIOCCHI M. (1993). Increased chemosensitivity to doxorubicin of intrinsically multidrug-resistant human colon carcinoma cells by prolonged exposure to verapamil. *Eur. J. Cancer*, **29A**, 1776–1778.

TSURUO T, IIDA H, NIJORI M, TSUKAGOSHI S AND SAKURAI Y. (1983). Circumvention of vincristine and adriamycin resistance in vitro and in vivo by calcium influx blockers. *Cancer Res.*, **43**, 2905–2910.

ZANETTE L, ZUCCHETTI M, FRESCHI A, ERRANTE D, TIRELLI U. AND D'INCALCI M. (1990). Pharmacokinetics of 4-demethoxydaunorubicin in cancer patients. *Cancer Chemother. Pharmacol.*, **25**, 445–448.


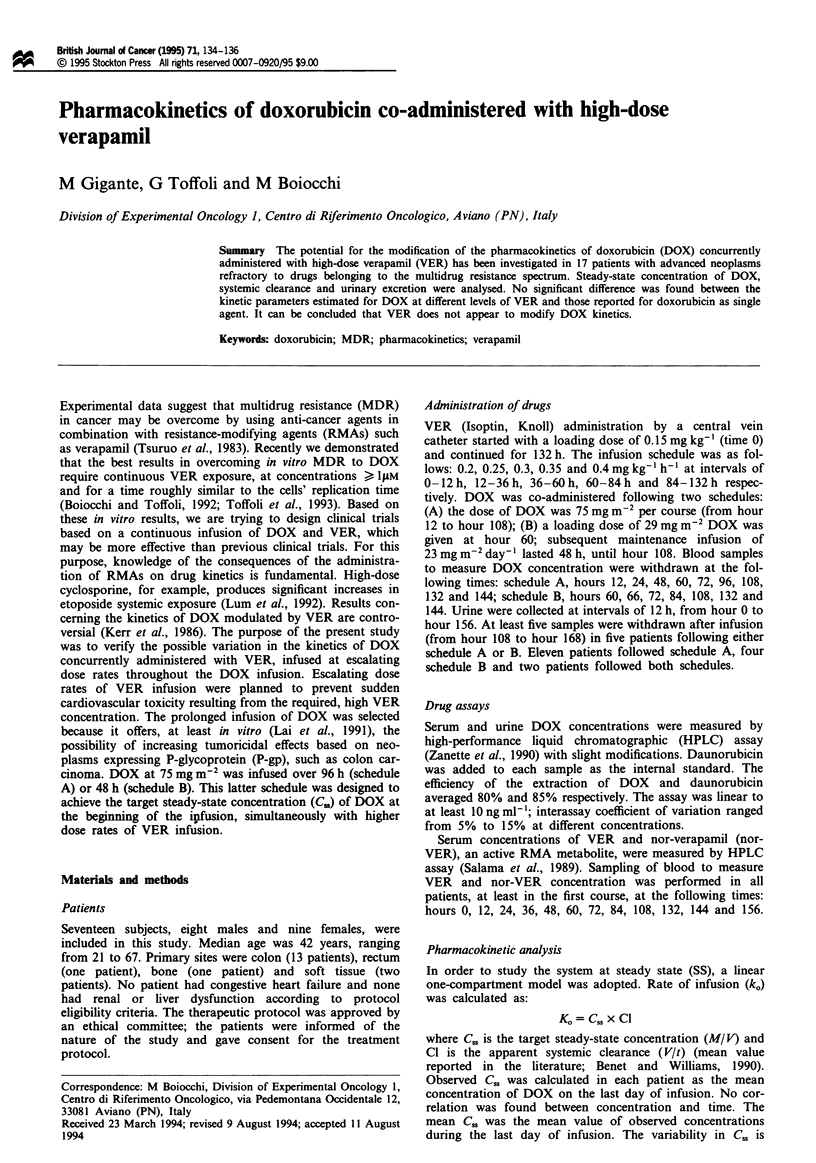

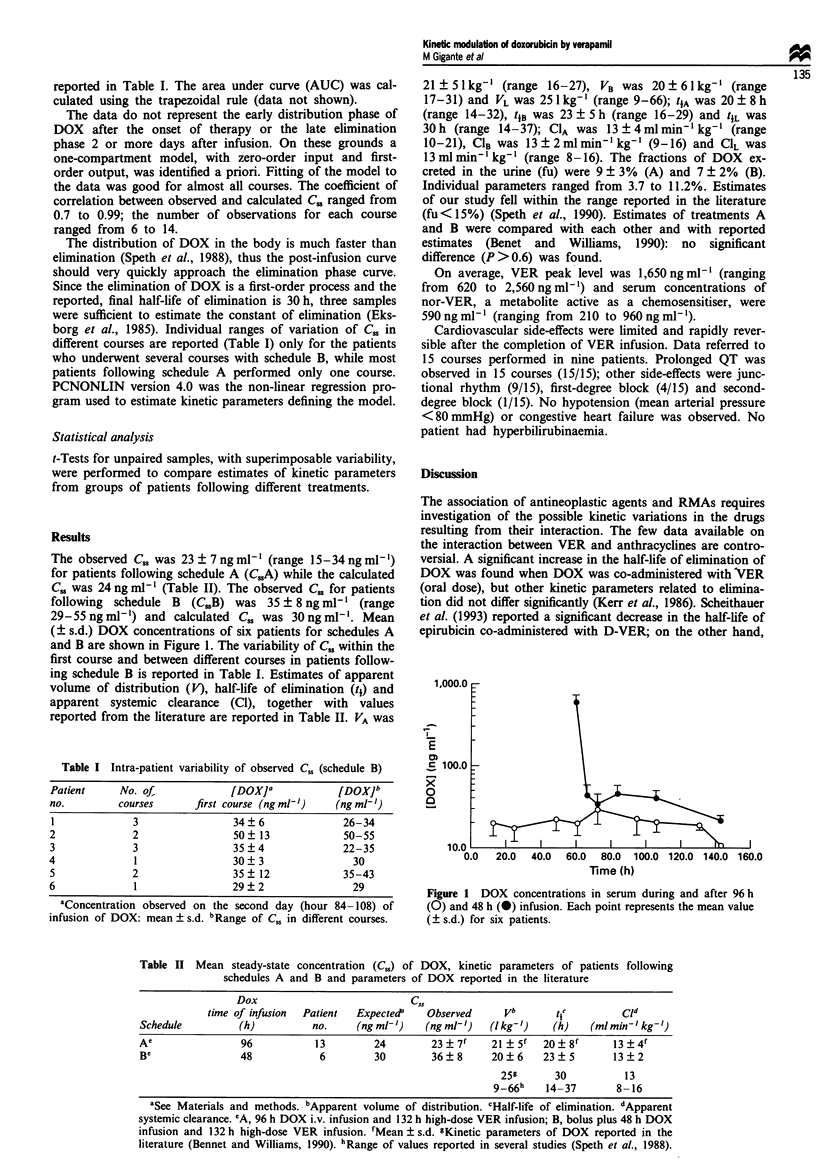

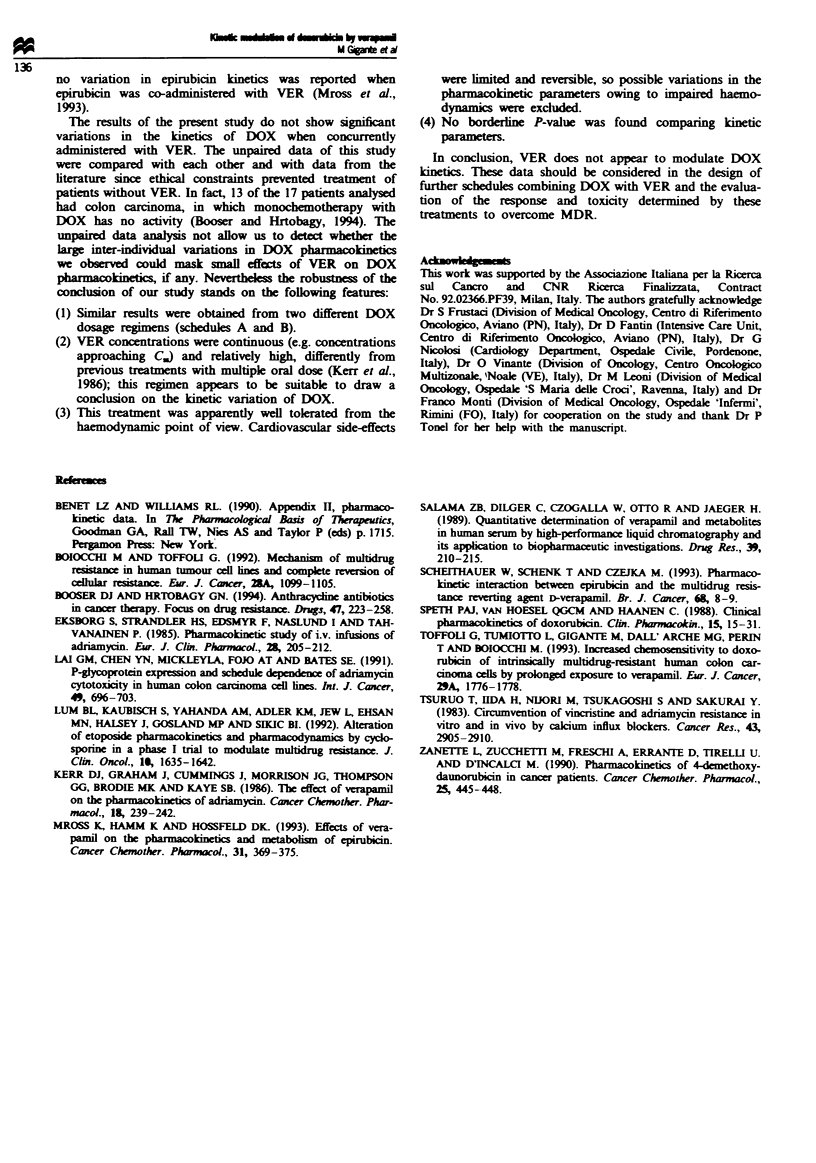


## References

[OCR_00365] Boiocchi M., Toffoli G. (1992). Mechanism of multidrug resistance in human tumour cell lines and complete reversion of cellular resistance.. Eur J Cancer.

[OCR_00368] Booser D. J., Hortobagyi G. N. (1994). Anthracycline antibiotics in cancer therapy. Focus on drug resistance.. Drugs.

[OCR_00373] Eksborg S., Strandler H. S., Edsmyr F., Näslund I., Tahvanainen P. (1985). Pharmacokinetic study of i.v. infusions of adriamycin.. Eur J Clin Pharmacol.

[OCR_00391] Kerr D. J., Graham J., Cummings J., Morrison J. G., Thompson G. G., Brodie M. J., Kaye S. B. (1986). The effect of verapamil on the pharmacokinetics of adriamycin.. Cancer Chemother Pharmacol.

[OCR_00378] Lai G. M., Chen Y. N., Mickley L. A., Fojo A. T., Bates S. E. (1991). P-glycoprotein expression and schedule dependence of adriamycin cytotoxicity in human colon carcinoma cell lines.. Int J Cancer.

[OCR_00384] Lum B. L., Kaubisch S., Yahanda A. M., Adler K. M., Jew L., Ehsan M. N., Brophy N. A., Halsey J., Gosland M. P., Sikic B. I. (1992). Alteration of etoposide pharmacokinetics and pharmacodynamics by cyclosporine in a phase I trial to modulate multidrug resistance.. J Clin Oncol.

[OCR_00402] Salama Z. B., Dilger C., Czogalla W., Otto R., Jaeger H. (1989). Quantitative determination of verapamil and metabolites in human serum by high-performance liquid chromatography and its application to biopharmaceutic investigations.. Arzneimittelforschung.

[OCR_00412] Speth P. A., van Hoesel Q. G., Haanen C. (1988). Clinical pharmacokinetics of doxorubicin.. Clin Pharmacokinet.

[OCR_00418] Toffoli G., Tumiotto L., Gigante M., Dall'Arche M. G., Perin T., Boiocchi M. (1993). Increased chemosensitivity to doxorubicin of intrinsically multidrug-resistant human colon carcinoma cells by prolonged exposure to verapamil.. Eur J Cancer.

[OCR_00422] Tsuruo T., Iida H., Nojiri M., Tsukagoshi S., Sakurai Y. (1983). Circumvention of vincristine and Adriamycin resistance in vitro and in vivo by calcium influx blockers.. Cancer Res.

[OCR_00430] Zanette L., Zucchetti M., Freshi A., Erranti D., Tirelli U., D'Incalci M. (1990). Pharmacokinetics of 4-demethoxydaunorubicin in cancer patients.. Cancer Chemother Pharmacol.

